# Rotating magnetocaloric effect and unusual magnetic features in metallic strongly anisotropic geometrically frustrated TmB_4_

**DOI:** 10.1038/s41598-018-29399-2

**Published:** 2018-07-19

**Authors:** Matúš Orendáč, Slavomír Gabáni, Emil Gažo, Gabriel Pristáš, Natalya Shitsevalova, Konrad Siemensmeyer, Karol Flachbart

**Affiliations:** 1 Institute of Experimental Physics, SAS, Watsonova Str. 47, 04001 Košice, Slovakia; 2 Institute of Physics, Faculty of Science, P. J. Safarik University, Park Angelinum Str. 9, 04154 Košice, Slovakia; 3 Institute for Problems of Materials Science, NASU, Krzhyzhanovsky Str. 3, 03680 Kiev, Ukraine; 4 Helmholtz-Zentrum Berlin, Glienicker Str. 100, 14109 Berlin, Germany

## Abstract

We have investigated the rotating magnetocaloric effect (R-MCE) of TmB_4_ - an anisotropic magnetic system with geometrical frustration of Shastry-Sutherland type. The R-MCE was obtained from detailed temperature dependencies of heat capacity in various magnetic fields of a single crystalline sample for crystal axes orientations *c* || *B* and *c* ⊥ *B*. The received results exhibit rather complex distributions of positive and negative entropy *ΔS(T, B)* and temperature *ΔT(T, B)* differences below and above *T*_*N*_ when the direction of the magnetic field changes between directions *c* || *B* and *c* ⊥ *B*. The calculated results were confirmed by direct R-MCE measurements which, moreover, show an interesting angular dependence of R-MCE in the ordered phase, which seems to be related with the change of the effective magnetic field along the *c* axis during sample rotation. Thus, our study presents a new type of magnetic refrigerant with a rather large R-MCE for low temperature magnetic refrigeration, and points to further interesting magnetic features in the ordered phase of this frustrated system.

## Introduction

The magnetocaloric effect (MCE) represents a magneto-thermodynamic phenomenon in which the temperature variation in magnetic material is caused by the change of external magnetic field^1–5^. It was also shown that geometrical spin frustration can significantly enhance the change of magnetic entropy in applied magnetic field and thus intensify the MCE^6,7^. On the MCE based magnetic refrigeration has attracted considerable attention as an alternative way of cooling, above all due to its energy efficiency and environmentally friendly way in comparison to conventional gas compression-expansion refrigeration. In order to improve the application possibilities of magnetic refrigeration, recently a novel rotating magneto-caloric effect (R-MCE) has been proposed and investigated^1,8–14^. In this case the MCE can be obtained by simply rotating the magnetic refrigerant in constant field instead of moving it in and out of the magnet. This rotary magnetic refrigeration can be used in case of strongly anisotropic magnetic materials and it seems to offer advantages in comparison with its conventional counterpart as it appears to be from the technical point of view more simple and compact.

In our contribution we present results of R-MCE investigations carried out on thulium tetraboride (TmB_4_), an anisotropic geometrically frustrated magnetic system. TmB_4_ belongs to the group of rare earth tetraborides (REB_4_) that crystallize in a tetragonal lattice^15,16^. As one of the three valence electrons of RE^3+^ ions goes to the conduction band, these tetraborides are good metals and the RKKY exchange interaction between magnetic ions is playing an important role. In case of TmB_4_ the magnetic Tm^3+^ ions have a 4f^12^ configuration with an angular momentum *J* = 6. In the mentioned tetragonal lattice the Tm ions lie in sheets perpendicular to the *c*-axis and can be within this (*a-b*) plane mapped onto the frustrated Shastry-Sutherland lattice, which can be viewed in terms of squares and equilateral triangles^17–21^. Between these Tm sheets there are planes of boron atoms grouped into B_6_ octahedra and dimer pairs. Crystal field effects at Tm^3+^ sites lift the degeneracy of the *J* = 6 multiplet. Consequently the ground state is a doublet *M*_*J*_ =  ±6 which induces a strong Ising-like magnetic anisotropy with magnetic moments of Tm ions oriented along the *c*-axis below its Néel temperature *T*_N_ = 11.7 K. In the ordered antiferromagnetic state the magnetization *M* for magnetic fields *B* || *c* reaches saturation *M*_*S*_ at about 4 T accompanied by magnetization plateaus at 1/2 *M*_*S*_ and 1/8 *M*_*S*_. On the other hand, for fields *B* ⊥ *c* the saturation of *M* is reached only at fields above 30 T. From this it follows that in magnetic fields up to about 4 T the magnetization along the *c*-axis is considerably higher than this in the perpendicular direction which is advantageous for the emergence of the R-MCE. Further details about the magnetic structure and other properties of TmB_4_ and related magnetic tetraborides^22–28^, as well as about the current theoretical approaches can be found elsewhere^29–33^.

Thus, TmB_4_ appears to be an interesting anisotropic frustrated magnetic system which literally invites to investigate its magnetocaloric properties, especially in the rotation version.

To study the R-MCE usually magnetization field dependencies *M*(*B, T*_0_) at various temperatures *T*_0_ for two perpendicular orientations (e.g. for *c* || *B* and *c* ⊥ *B*) are measured, on the base of which the entropy difference *ΔS*(*T, B*) related with these two orientations is calculated (see e.g. ref.^9^). According to *ΔS* the corresponding adiabatic temperature change *ΔT*(*T, B*), which denotes the temperature difference between the state with lower entropy (in case of TmB_4_ for *B* || *c*) and this with a higher entropy (for TmB_4_ when *B* ⊥ *c*) can be estimated. In this method, however, usually a constant heat capacity of the investigated material is used, which can often lead to considerable errors in the *ΔT* estimation. Therefore, in our case the R-MCE investigation of TmB_4_ was based on detailed heat capacity *C*(*T, B*_0_) measurements in a wide *T* and *B* range (for directions *B* || *c* and *B* ⊥ *c*) from which the entropy difference *ΔS* and temperature *ΔT* were calculated using the method described in ref.^34^. The received results were verified experimentally by direct *ΔT*_*exp*_(*T, B*) measurements, and analyzed and interpreted by complementary angular-dependent magnetization measurements.

## Materials and Methods

Single crystals of TmB_4_ were grown by an inductive, crucible-free zone melting method. The residual resistivity ratio of investigated samples was larger than 100, documenting their high quality. For heat capacity experiments an oriented sample with approximate dimension 1 × 1 × 0.5 mm^3^ was cut. The same sample, or a part of it, was used also for other measurements performed within this work. *C*(*T, B*) measurements in the temperature range 2–60 K and in magnetic field up to 4.8 T were performed using a commercial Quantum Design PPMS system with a built-in relaxation method. For every experimental point the temperature and magnetic field were fixed. From *C*(*T, B*) results the entropy *ΔS* and temperature *ΔT* changes were determined. To measure *ΔT*_*exp*_(*T, B*) (and its angular dependence) directly, a special home-made rotary calorimeter was constructed (a more detailed description of the calorimeter can be found in part “Rotating magnetocaloric effect of TmB_4_”). Because of the high magnetic anisotropy of TmB_4_ and from this resulting (especially in high magnetic fields) large torque ***τ*** = ***M*** × ***B***, where ***M*** denotes the magnetization, the sample (a relatively large one ∼10 mg, to receive a high *ΔT* signal) was fixed to the calorimeter by means of a bulky amount of glue (GE Varnish). The rather robust (6 × 6 × 0.3 mm^3^) sapphire calorimeter equipped with a ruthenium oxide thermometer was fixed to the temperature stabilized platform by four fishing lines (*ϕ* ≈ 0.1 mm). The corresponding measurements were performed in a Quantum Design Physical Property Measurement System (PPMS) equipped with a rotator option. Measurements of the empty (without sample) calorimeter with glue have shown that its total heat capacity below 20 K is about 10 times bigger than that of the investigated sample which led to the fact that the experimentally observed *ΔT*_exp_ values appear to be considerably smaller than those calculated (and expected) from above mentioned heat capacity measurements. The associated angular dependence of magnetization at various temperatures and magnetic fields was determined by a horizontal sample rotator option (M101C) of the Quantum Design Magnetic Property Measurement System (MPMS) which enabled to rotate the TmB_4_ sample with respect to magnetic field orientation.

### Anisotropy of TmB_4_ above *T*_*N*_

**Figure 1:**
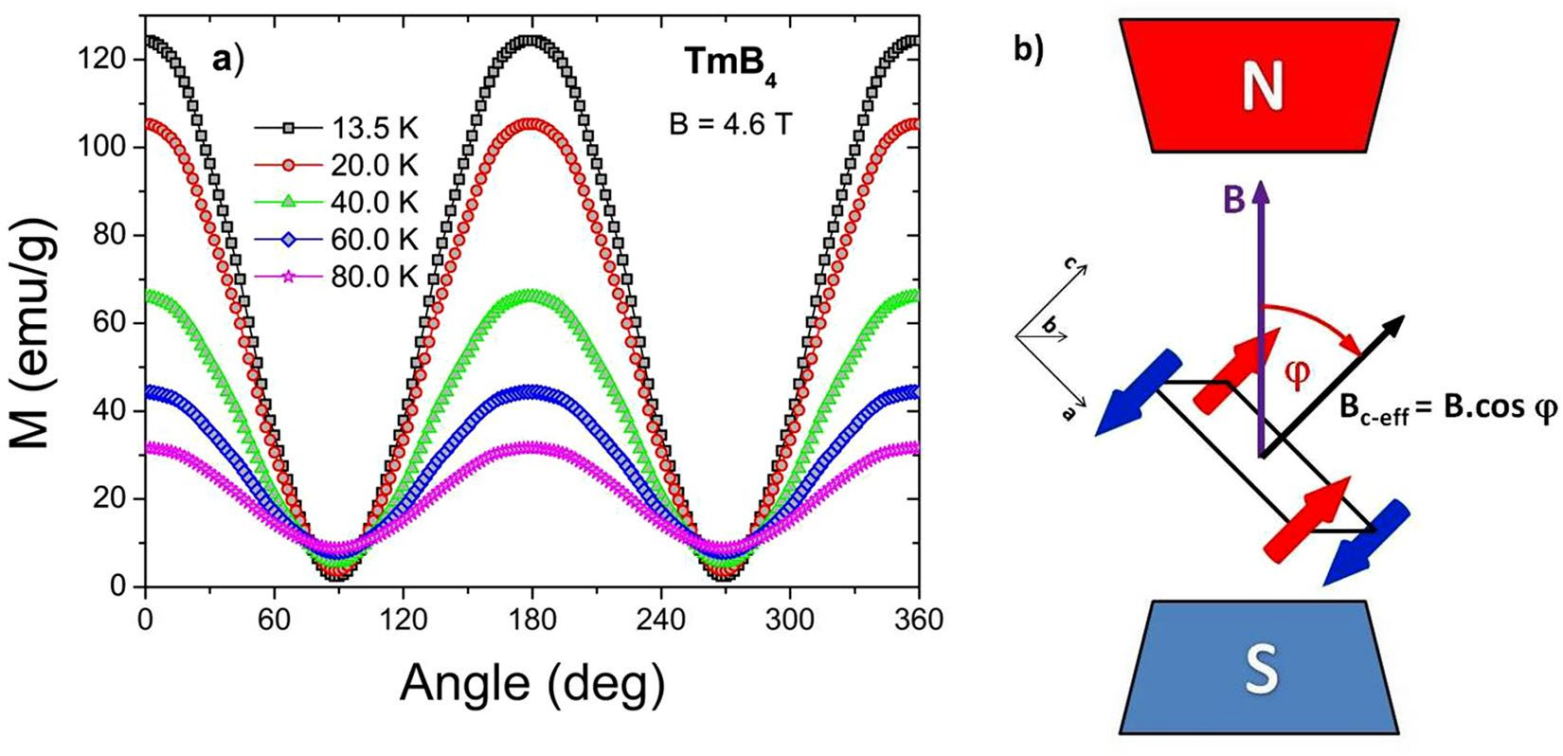
(**a**) Angular dependence of magnetization *M*(*φ*) at various temperatures above *T*_*N*_ in field of 4.6 T. The anisotropy of *M*(*φ*) shows a harmonic/sinusoidal behaviour and its magnitude decreases with increasing temperature. (**b**) Sample rotation with respect to the field.

To study the R-MCE of TmB_4_, it is necessary to know the anisotropy of its magnetic properties in the paramagnetic state above *T*_*N*_. In Fig. 1a the angular dependencies of magnetization *M*(*φ*) at various temperatures is displayed. As can be seen, these dependencies are harmonic/sinusoidal with maxima at *φ* = 0° (when *c* || *B*) and minima at *φ* = 90° (when *c* ⊥ *B*, in fact *c* ⊥ *B* corresponds in our case always to (110) direction). At *T* = 13.5 K and *B* = 4.6 T the ratio between the maximum *M*_*max*_ (*c* || *B*) and minimum *M*_*min*_ (*c* ⊥ *B*) has a value of *M*_*max*_/*M*_*min*_ ≈ 50! This very high value confirms that TmB_4_ is strongly anisotropic also in the paramagnetic phase, above all close above *T*_*N*_, and that this material should be suitable for its use in rotating magnetic calorimetry. On the other hand, the high anisotropy also shows that in TmB_4_ (at least close above *T*_*N*_) are the magnetic moments of Tm ions factually exclusively oriented parallel to the *c* - axis. Therefore, it can be assumed that sample rotation in magnetic field (which is equivalent to field rotation with respect to the sample) causes only a change of the field amplitude along the *c* - direction (see Fig. 1b). In such a case the field along the *c* - direction is *B*_*c-eff*_ = *B*.cos*φ*, where *φ* is the angle between the applied field *B* and the *c* - axis during rotation. Thus, the highest magnetisation in this anisotropic system is observed when *B* || *c* (*φ* = 0°) and the lowest one when *B* ⊥ *c* (*φ* = 90°). With increase of temperature the *M*_*max*_/*M*_*min*_ ratio gradually decreases (see Fig. 1a) which points to the fact that the R-MCE is most pronounced in the temperature region not too far above *T*_*N*_.

### Rotating magnetocaloric effect of TmB_4_

**Figure 2:**
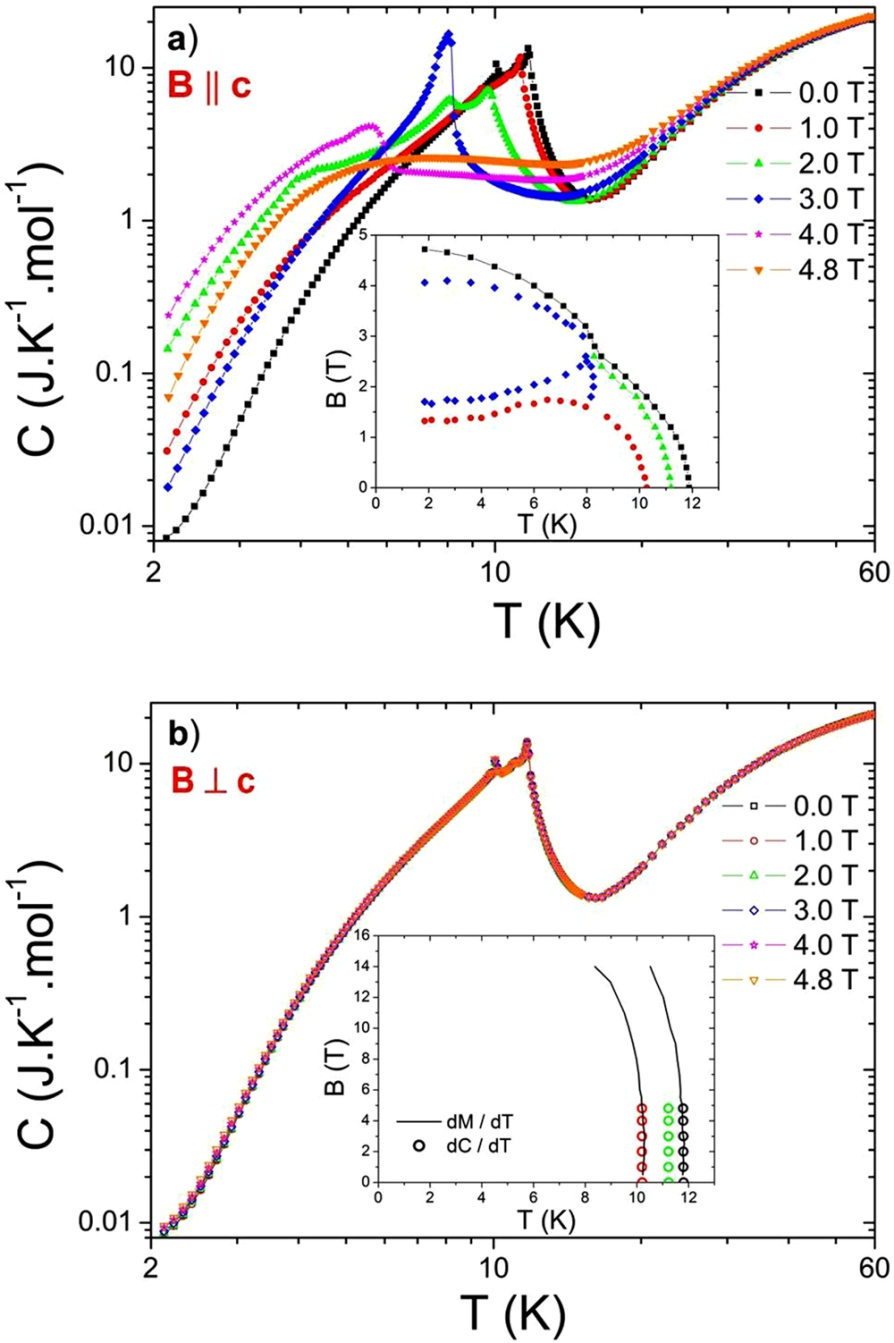
Temperature dependencies of heat capacity *C*(*T, B*_0_) of TmB_4_ in various magnetic fields for field orientations *B* || *c* (**a**) and *B* ⊥ *c* (**b**). One can see that in fields up to 4.8 T the *C*(*T*) results for *B* ⊥ *c* practically overlap. The inserts show the corresponding *B-T* phase diagrams which agree with those obtained from magnetization measurements^17,18^ (for the case *B* ⊥ *c* denoted by solid lines).

The obtained results of the temperature dependencies of heat capacity in various magnetic fields *C*(*T, B*_0_) for field orientations *B* || *c* and *B* ⊥ *c* (for both orientations the same sample was used) are shown in Fig. 2. In this case the *C*(*T, B*_0_) dependencies were measured starting from the highest temperature towards the lowest, then the sample was warmed up quickly back to the highest temperature and subsequently the field was changed (increased). Whereas for *B* ⊥ *c* are the *C*(*T, B*_0_) dependencies in fields up to 4.8 T (within the measurement error) practically identical, for *B* || *c* pronounced field dependent changes were observed (see Fig. 2) which is in accordance with magnetization measurements on this compound (see e.g. refs^17,18^). The corresponding *B-T* phase diagrams received from *C*(*T, B*_0_) dependencies, which agree with those based on magnetization measurements^17,18^, are shown as inserts of Fig. 2 (the higher “*B*” values of phase boundaries in phase diagrams are associated with the fact that in this case the demagnetization factor of the used sample was not taken into account, the reason is related to further experiments in which the sample was rotated). The reason why the *C*(*T, B*_0_) measurements were performed only up to 4.8 T was associated with the fact that above this value the torque acting on the sample (fixed on calorimeter) already started to rotate it (or tear it from the calorimeter).

Based on these *C*(*T, B*_0_) dependencies corresponding entropy dependencies *S*(*T, B*) for both directions (*B* || *c* and *B* ⊥ *c*) have been calculated using the relation (1):

1 $$S(T,\,B)={\int }_{0}^{T}\frac{C(T,\,B)}{T}dT$$

**Figure 3:**
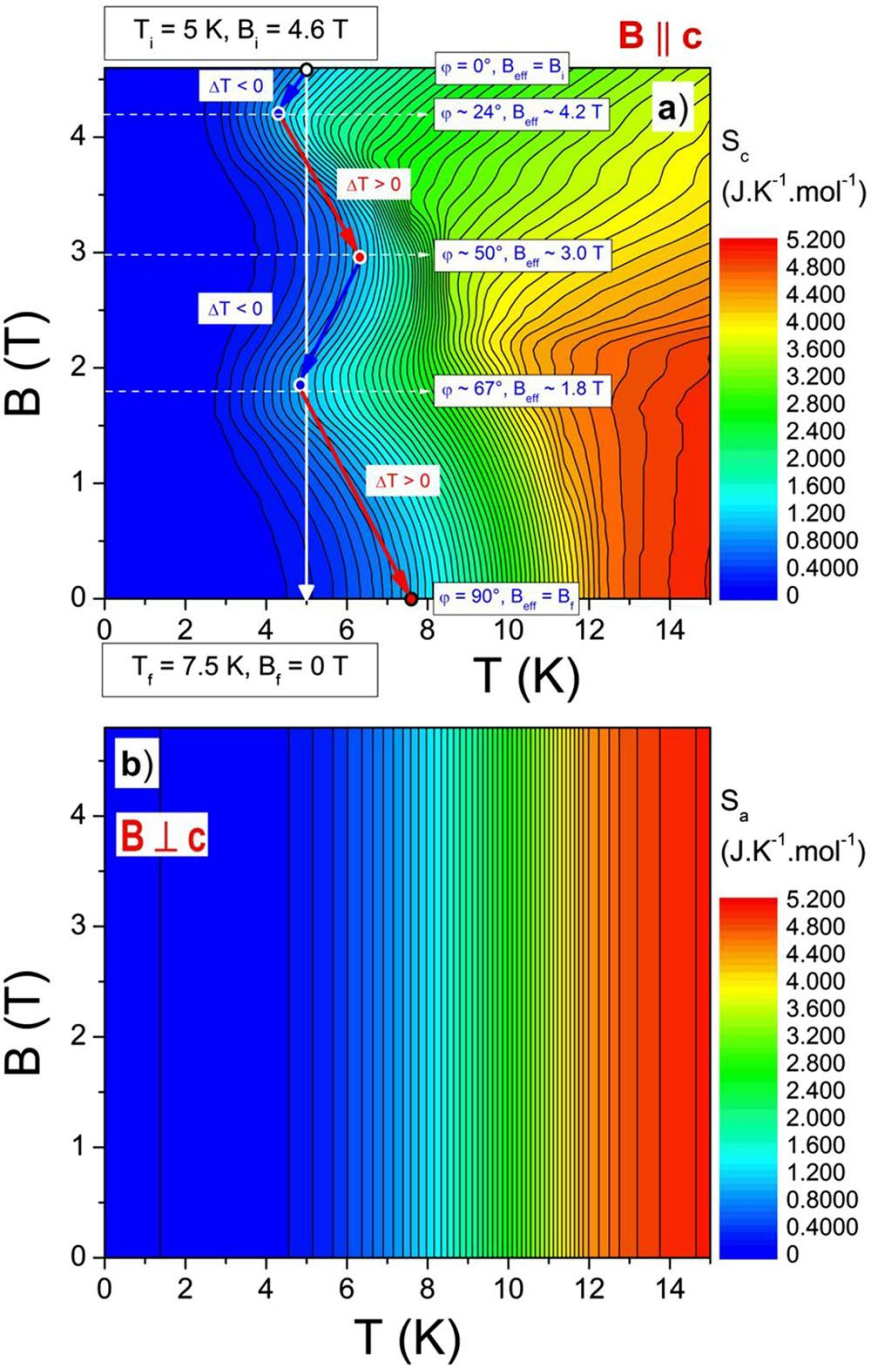
Entropy distributions for *B* || *c* (**a**) and *B* ⊥ *c* (**b**) calculated from temperature dependencies of heat capacity *C*(*T, B*_0_). Note that the contours of entropy distribution for *B* || *c* display rather well the *B-T* phase diagram of TmB_4_. The blue and red arrows in (**a**) indicate the temperature changes *ΔT* (cooling and heating) related with the isentropic course of entropy *S*_*c*_ during sample rotation in the ordered phase (this phenomenon is discussed in more detail in part “Special magnetic features of TmB_4_ below *T*_*N*_”).

To perform these calculations from *T* = 0 a linear extrapolation of heat capacity towards zero temperature was used, and the entropy at *T* = 0 was for all fields and sample orientations set to zero. But it was also shown that if the linear extrapolation of *C*(*T, B*_0_) dependencies below 2 K was replaced by a realistic Schottky contribution coming from thulium nuclei which was in detail down to 20 mK investigated in ref.^35^, the obtained *S*(*T*, *B*) differences at *T* > 3 K were irrelevant. The calculated distributions of entropy are shown in Fig. 3. Note that even if the entropy calculation (see eq. 1) smoothes the heat capacity anomalies, the layout of entropy for *B* || *c* displays rather well the *B-T* phase diagram of TmB_4_ for this direction.

**Figure 4:**
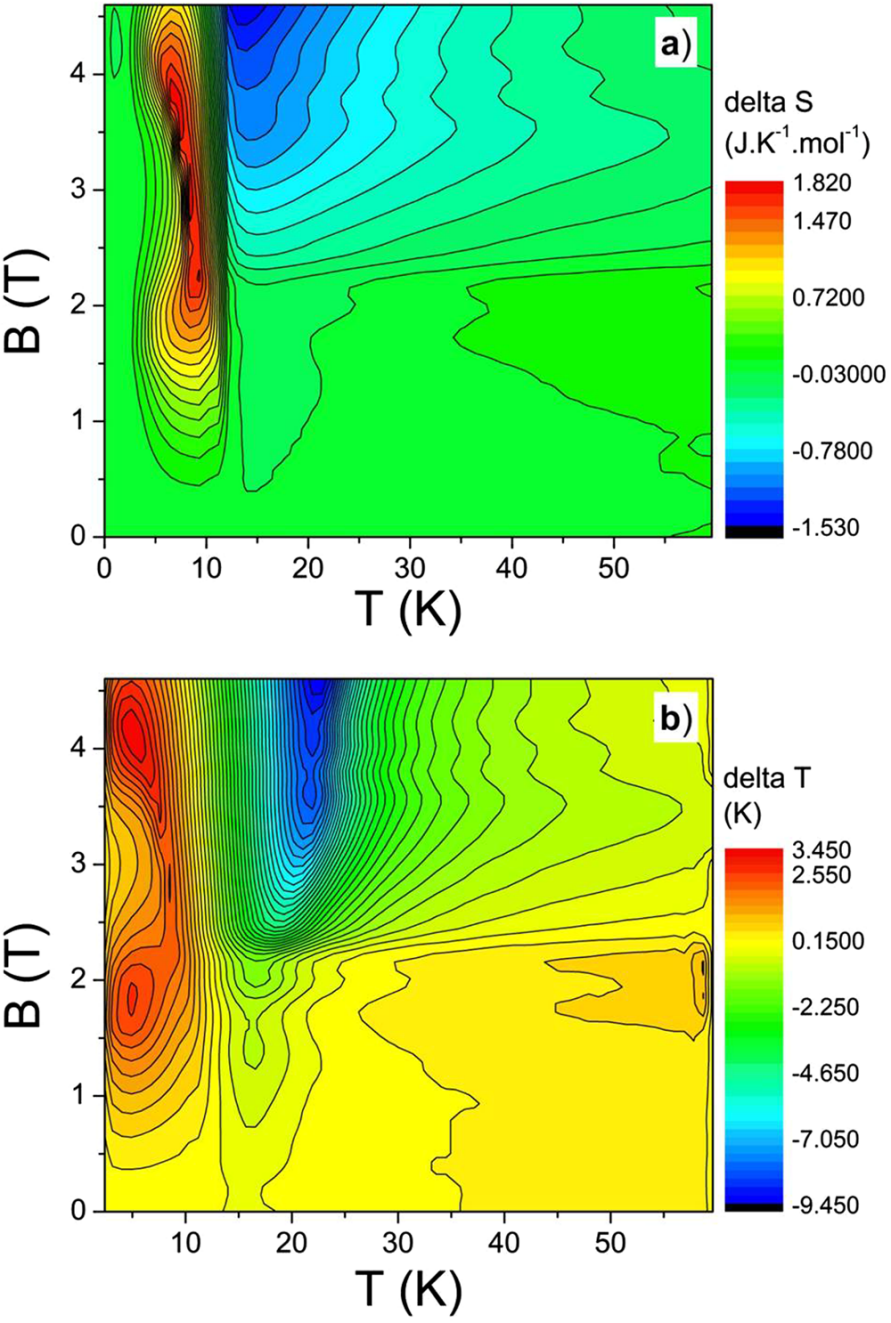
Distribution of the entropy difference *ΔS*(*T, B*) which arises during rotation of TmB_4_ from *B* || *c* to *B* ⊥ *c* in fields up to 4.6 T (**a**) and layout of the corresponding temperature difference *ΔT*(*T, B*) determined from *S*(*T, B*) distributions in Fig. 3 using eq. (2) (**b**).

The difference between entropies *ΔS* for *B* || *c* and for *B* ⊥ *c* is shown in Fig. 4a. It exhibits a “heating” hill in the ordered phase with a summit around 9 K and a wide “cooling” depression in the paramagnetic phase around 15 K which is deepening with the increase of magnetic field. Based on received *S*(*T, B*) distributions for *B* || *c* and for *B* ⊥ *c* (Fig. 3) the corresponding temperature change *ΔT* between directions *B* || *c* and *B* ⊥ *c* can be determined. We would like to note that in the case when *S* distributions are calculated from magnetization data the corresponding *ΔT* is usually estimated using a constant (average) heat capacity *C*(*T, B*) value, which can lead to significant errors if the heat capacity in the particular region changes markedly (see Fig. 2). These errors can be avoided if entropy calculations are based on heat capacity measurements. In this case, the isentropic temperature difference *ΔT*(*T, B*) for every (*T*, *B*) point can be calculated using the relation (2):

2 $${\rm{\Delta }}T(T,\,B)={T}_{B\perp c}(B,\,S)-{T}_{B||c}(B,\,S),$$

where *T*_*B*⊥*c*_(*B*, *S*) and *T*_*B|| c*_(*B*, *S*) are the temperatures at a certain (*B, S*) point (based on Fig. 3) which has the same *B* and *S* values both for the *B* ⊥ *c* and the *B* || *c* orientations (e.g. at starting point for *B* || *c*, *B* = 4.5 T and *T*_*B|| c*_ = 15 K the entropy is *S* = 3.58 JK^−1^ mol^−1^, after adiabatic rotation in the same field to *B* ⊥ *c* the final temperature is *T*_*B*⊥ *c*_ = 11.5 K, i.e. *ΔT* ≈ −3.5 K). The resulting *ΔT*(*T, B*) dependence is illustrated in Fig. 4b. Even if the *ΔT*(*T, B*) dependence is similar to the *ΔS* one, due to the marked *C*(*T, B*_0_) distribution is the *ΔT* layout different. It exhibits a large cooling region above *T*_*N*_ (around 20 K and in fields above 2 T) in which the temperature of TmB_4_ during the same rotation decreases by more than 9 K (this cooling procedure is analogous to the conventional demagnetisation process in the paramagnetic region). But, there is also a positive (warming up) area below *T*_*N*_ (around 5 K at 1.8 T and 4.2 T) where the temperature increases by more than 2.5 K when the sample is rotated from *B* || *c* to *B* ⊥ *c* and which is related with heating at magnetic reversal in the ordered state. These results thus exhibit an interesting and rather peculiar R-MCE distribution in this strongly anisotropic frustrated metallic system. Moreover, our results show that estimations of the magnetocaloric effect based on magnetization data (and on an usually average heat capacity value) can lead to inaccuracies in Δ*T* determination, especially at the ordering temperature where heat capacity anomalies occur. However, this does not apply to the main MCE cooling region above *T*_*N*_.

The relevant refrigerant capacity (*RC*) was estimated according to refs^9,34^ using the expression:

3 $$RC={\int }_{{T}_{1}}^{{T}_{2}}|{\rm{\Delta }}S|dT$$

where *T*_1_ and *T*_2_ are the temperatures corresponding to sides of the half-maximum |*ΔS*| peak value. For TmB_4_ the “heating” refrigerant capacity for the positive *ΔS* peaks at *B* = 1.8 T and 4.2 T is *RC* ≈ 37.20 J/kg and 32.02 J/kg, respectively. On the other hand the “cooling” refrigerant capacity for the negative *ΔS* depression at *B* = 4.6 T is *RC* ≈ 87.51 J/kg, which is comparable with values of other R-MCE materials (see e.g. ref.^9^).

**Figure 5:**
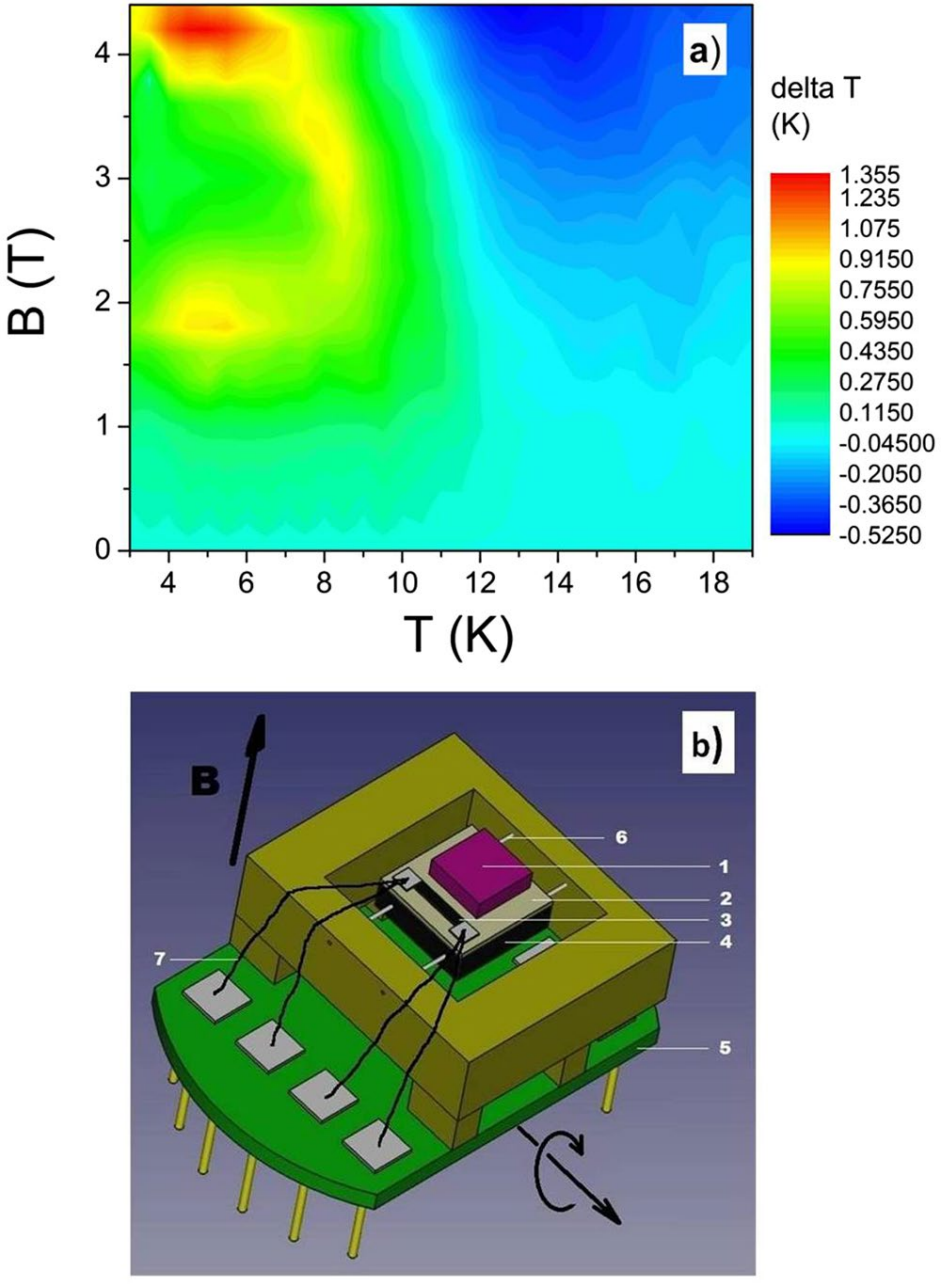
Distribution of the experimentally obtained R-MCE depicted as temperature difference *ΔT*_*exp*_(*T, B*) when starting at *T* and *B* values the single crystal of TmB_4_ is rotated from *B* || *c* to *B* ⊥ *c* (**a**). The difference between this distribution and that in Fig. 4b comes mainly from the large heat capacity of the rotary calorimeter which was used to determine *ΔT*_*exp*_(*T, B*). (**b**) - rotary calorimeter: 1 - sample, 2 - sapphire calorimeter, 3 - thermometer, 4 - Vespel support, 5 - PPMS puck, 6 - silon rope, 7 - bronze leading wire.

In order to verify our predictions based on *C*(*T, B*) measurements we have performed direct R-MCE measurements using a rotary calorimeter. The distribution of the experimentally obtained temperature difference *ΔT*_*exp*_(*T, B*) is displayed in Fig. 5 (*ΔT*_*exp*_(*T, B*) represents the temperature change of the system “sample plus calorimeter”). This distribution is similar to that of *ΔT*(*T, B*) in Fig. 4b (determined from heat capacity measurements). The differences between absolute values of *ΔT*_*exp*_(*T, B*) and *ΔT*(*T, B*) are associated with the rather large heat capacity of the rotary calorimeter used for determination of *ΔT*_*exp*_(*T, B*) (as mentioned in section Materials and methods, the heat capacity of the calorimeter was about 10 times larger than this of the used sample). On the other hand, the difference between *ΔT*_*exp*_(*T, B*) and *ΔT*(*T, B*) layouts (e.g. the cooling minimum of *ΔT*_*exp*_(*T, B*) is observed at lower temperatures than this of *ΔT*(*T, B*)) is most probably associated with the fact that the sharp *C*(*T*, *B*) changes near *T*_N_ (Fig. 2) which determine the *ΔT*(*T, B*) values, are in case of *ΔT*_*exp*_(*T, B*) estimation (due to the rather large heat capacity of the rotary calorimeter) considerably reduced.

**Figure 6:**
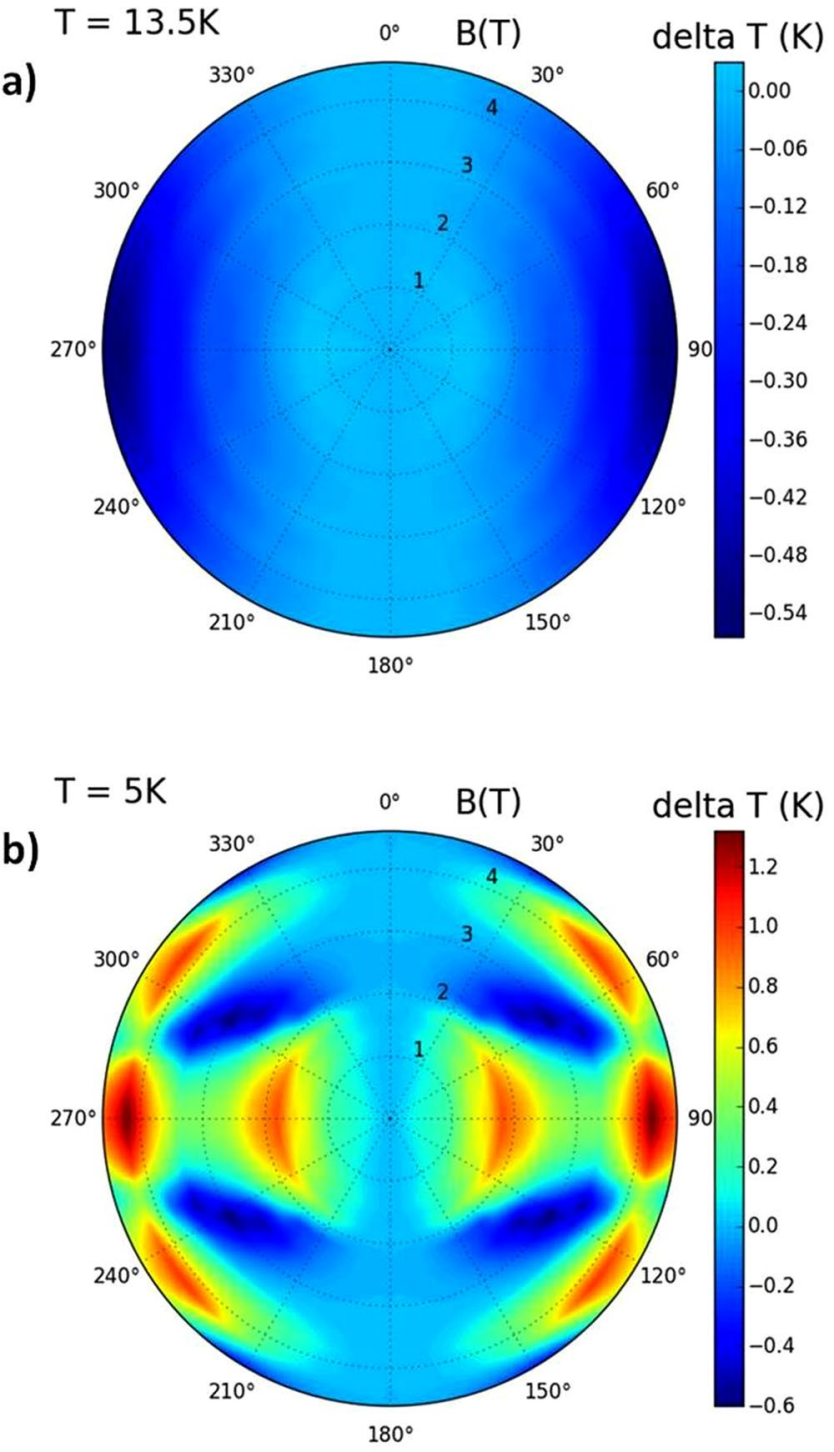
Layout of the experimentally determined angular dependence of the R-MCE at 13.5 K (**a**) and at 5 K (**b**) shown in polar coordinates as a temperature difference *ΔT*_*exp*_(*φ, B*) of the calorimeter (including sample) during rotation between *c* || *B* (*φ* = 0° or 180°) and *c* ⊥ *B* (*φ* = 90° or 270°) in magnetic fields *B*.

Results of the detailed angular dependence of direct R-MCE investigations (i.e. measurements of *ΔT*_*exp*_(*φ, T, B*)) using the home-made calorimeter, which allowed to rotate the TmB_4_ sample smoothly between *c* || *B* and *c* ⊥ *B*, are for various magnetic fields shown in Fig. 6. Above *T*_N_ (e.g. at 13.5 K) such a rotation leads (as expected) to continuous cooling which intensifies with increasing field. However, the angular dependence of R-MCE below *T*_N_ (e.g. at 5 K) shows a rather complex behaviour, especially in higher magnetic fields. As can be seen, with increasing *φ* the heating process (due to magnetic reversal) is not monotonous, and except the expected peak at *φ* = 90° (when the sample was rotated from *B* || *c* to *B* ⊥ *c*) it exhibits an anomaly also around *φ* ≈ 60°. The reason for this observation is discussed in the next section.

### Special magnetic features of TmB_4_ below *T*_*N*_

**Figure 7:**
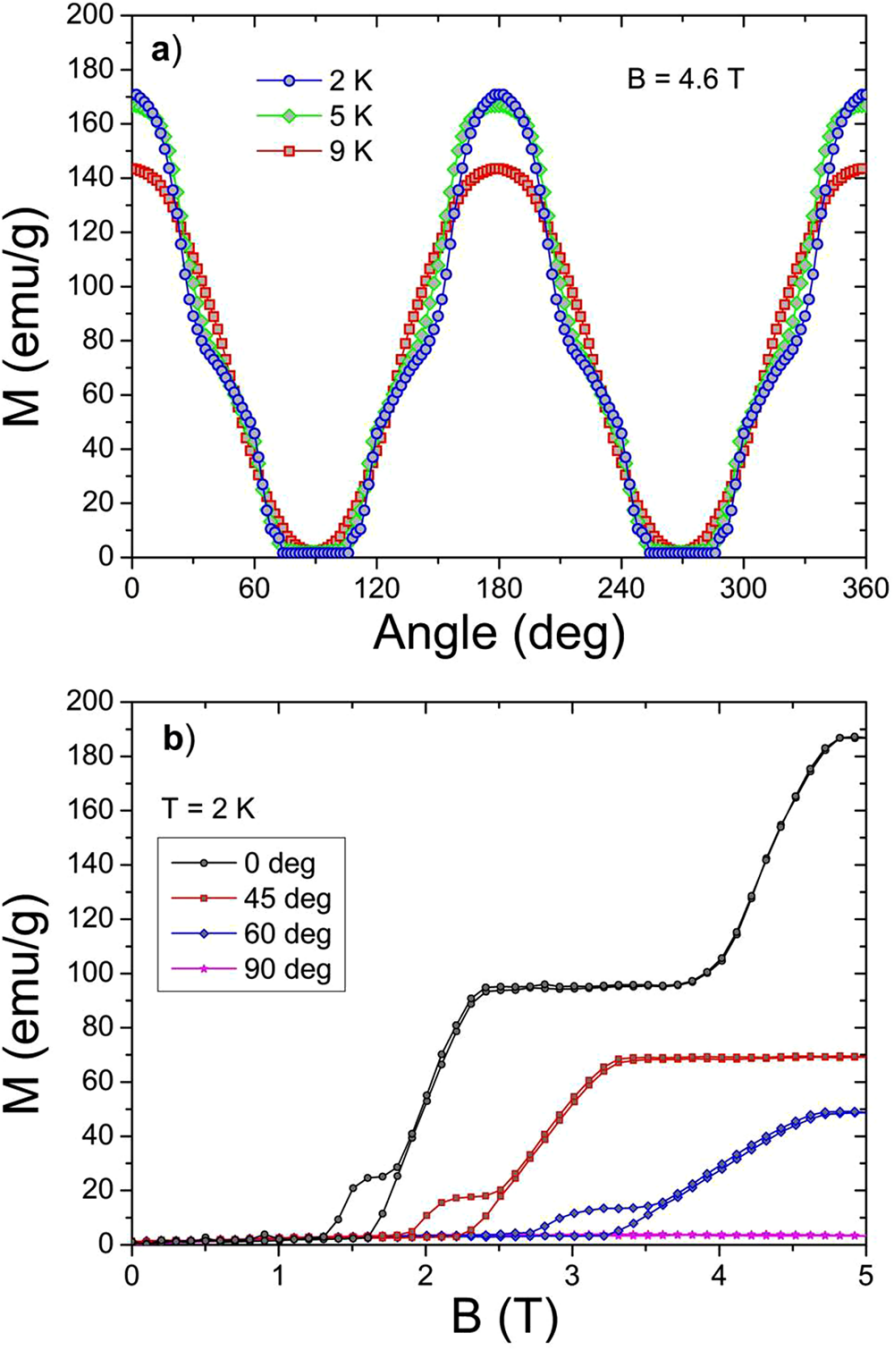
Angular dependence (anisotropy) of magnetization *M*(*φ*) below *T*_*N*_ in field of 4.6 T and at various temperatures (**a**). (**b**) Field dependencies of magnetisation at various *φ* angles.

To investigate the observed heating anomaly of R-MCE (at *φ* ≈ 60°) in the ordered state more in detail, angular dependent magnetization measurements at temperatures below *T*_*N*_ and in various magnetic fields were performed. Under these conditions *M*(*φ*) does not anymore exhibit a sinusoidal dependence when the sample is rotated from *φ* = 0° (*c* || *B*) to *φ* = 90° (*c* ⊥ *B*), but a rather complicated course (see Fig. 7a) which depends both on magnetic field and temperature. Nevertheless, also in this case the angular dependence shows their maxima at *φ* = 0° and minima at *φ* = 90°. At *T* = 2 K and *B* = 4.6 T the ratio between the magnetisation maximum *M*_*c*_ and minimum *M*_*a*_ has a value of *M*_*max*_/*M*_*min*_ ≈ 40. This value confirms the very high anisotropy of TmB_4_ in the ordered phase^17^. Due to this (as in the paramagnetic phase) also in the ordered phase one can be expect that in TmB_4_ are the magnetic moments in fields up to 5 T are exclusively oriented parallel with the c axis. Therefore, it can be by analogy (as in the paramagnetic phase) assumed that the sample rotation in magnetic field manifests itself as a *B*_*c-eff*_ = *B*.cos*φ* change of the field along the *c* - direction (see Fig. 1b). In this way, e.g. at 5 K and in magnetic field of 5 T, during rotation from *φ* = 0° to *φ* = 90° the magnetic field gradually passes through all ordered magnetic phases of TmB_4_, the half plateau (ferrimagnetic) phase, the fractional plateau phase and the Néel phase (detailed information about magnetic phases of TmB_4_ can be found e.g. in refs^19,24^). On the other hand, taking into account the observed very large anisotropy in the ordered phase, it appears surprising that complex structures are expected to arise at magnetic domain walls as suggested in ref.^24^.

Taking into account the above suggestion that upon rotation the effective field *B*_*c-eff*_ along the *c* axis changes, the experimentally observed angular dependences of R-MCE in the ordered phase (Fig. 6b) can be linked with the isentropic course of entropy *S*_*c*_ (Fig. 3a, see the course indicated by arrows). Starting e.g. with *S*_*c*_ at *T* = 5 K, *B* = 4.6 T and reducing *B*_*c-eff*_ along the line *S*_*c*_ = const., one can see two repeating cooling (blue arrows) and two heating intervals (red arrows), which is in agreement with *ΔT*_*exp*_(*φ, B*) behaviour in Fig. 6b (if one at 4.6 T changes *φ* from 0° to 90°). The same applies for *T* = 5 K, *B* = 3 T where one cooling and one heating interval can be observed, etc. But, on the other hand, this method of *ΔT* estimation in Fig. 3a cannot be straightforwardly applied also at higher temperatures, e.g. above *T*_*N*_. The reason is that changes of *ΔT*_*exp*_(*φ, B*) in Fig. 6b are changes in the system “sample plus calorimeter”, whereas Fig. 3a shows entropy distributions (and related *ΔT* changes) of the sample only. And, as the heat capacity of the dielectric calorimeter depends on temperature as ∼*T*^3^, it can be expected that conclusions and comparisons based on Figs 3a and 6b will diverge with increasing *T*.

However, one has to take into account that upon sample rotation and the corresponding field change of *B*_*c-eff*_ also the magnetic structure itself within relevant magnetic phases may change. Thus, e.g. in the half plateau (ferrimagnetic) phase with the decrease of *B*_*c-eff*_ not all magnetic moments will remain oriented in the field direction, but a part of them will start to flip and point into the opposite direction. And this applies probably also for the fractional plateau phase. This fact is reflected e.g. in field dependencies of magnetisation at various *φ* angles (Fig. 7b). There one can see that with increasing angle *φ* not only the fractional plateau phase and half plateau phases become (as expected) shifted to higher *B* (as the field along *c* - direction changes as *B*_*c-eff*_ = *B*.cos*φ*, higher fields are needed to reach the plateaus), but also the values of magnetization magnitudes in plateau regions become reduced (as a result of increasing spin flips in the opposite direction). These results point to further interesting magnetic features in the ordered phase of this anisotropic frustrated system, and it will be interesting to investigate e.g. how the phase diagram and the plateaus will change depending on angle *φ*.

## Conclusions

We have shown that TmB_4_ exhibits very strong magnetic anisotropy both in the ordered as well as in the non-ordered/paramagnetic phase. Based on this fact and on detailed temperature dependencies of heat capacity in various magnetic fields *C*(*T, B*_0_) for crystal axes orientations *c* || *B* and *c* ⊥ *B* we have determined the R-MCE of TmB_4_ - a magnetic system with a geometrical frustration of Shastry-Sutherland type. The received R-MCE results exhibit a significant cooling effect above *T*_*N*_ and a rather complex *ΔT* distributions of cooling and heating below *T*_*N*_. These results were confirmed experimentally by direct *ΔT*_*exp*_ measurements which have in addition shown an interesting angular R-MCE dependence in the ordered phase. As angle-dependent magnetization measurements just above and below *T*_*N*_ have shown, are the magnetic moments in TmB_4_ oriented only parallel to the *c* - axis. Therefore, it can be assumed that the sample rotation in magnetic field manifests itself as a change of the field amplitude along *c* - direction. From this follows that the experimentally observed angular dependencies of R-MCE in the ordered phase can be explained by transitions through different magnetic phases upon sample rotation. Thus, our study shows TmB_4_ as an interesting anisotropic system with geometrical frustration which is suitable for R-MCE at low temperatures. Moreover, results in the ordered phase point to further interesting questions related e.g. to the angular dependence of its magnetic properties.
